# Preliminary Study of the Rhenium Addition on the Structure and Mechanical Properties of Tungsten Heavy Alloy

**DOI:** 10.3390/ma14237365

**Published:** 2021-11-30

**Authors:** Paweł Skoczylas, Mieczysław Kaczorowski

**Affiliations:** Department of Mechanics and Armament Technology, Faculty of Mechanical and Industrial Engineering, Warsaw University of Technology, Narbutta 85, 02-524 Warsaw, Poland; mieczyslaw.kaczorowski@gmail.com

**Keywords:** tungsten heavy alloy, rhenium addition, microstructure, mechanical properties, liquid phase sintering

## Abstract

The results of structure and mechanical property investigations of tungsten heavy alloy (THA) with small additions of rhenium powder are presented. The material for the study was prepared using liquid phase sintering (LPS) of mixed and compacted powders in a hydrogen atmosphere. From the specimens, the samples for mechanical testing and structure investigations were prepared. It follows from the results of the microstructure observations and mechanical studies, that the addition of rhenium led to tungsten grain size decreasing and influencing the mechanical properties of W-Ni-Fe-Co base heavy alloy.

## 1. Introduction

**Figure 1:**
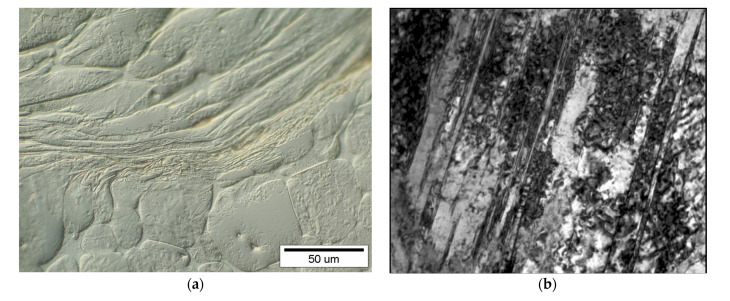
The example of ASB in the tungsten heavy alloys penetrator: (**a**) metallographic photo, (**b**) transmission electron micrograph (TEM) area × 40.000.

Tungsten heavy alloys (THA) belong to the group named weight heavy alloys (WHA), which are characterized with very high density and good mechanical properties [1,2,3,4,5,6,7,8,9,10,11,12,13,14,15,16]. One of the special applications is kinetic energy penetrators (KEP) [17,18,19,20,21,22,23,24]. Up to today, these penetrators were commonly made of depleted uranium (DU), which is technologically easier and better from a ballistic point of view (depth of penetration). However, because of its radioactivity, DU penetrators are successively replaced with tungsten heavy alloys; because of the very high melting point of tungsten (3420 °C), the powder metallurgy (PM) had to be used. Among a few methods of sintering, liquid phase sintering (LPS) is most often used [1,2,3,7,15,25,26,27,28]. As was already said, DU penetrators exceed the THA with depth of penetration, which is caused by so-called “self-sharpening” effects of ascribed adiabatic shear bands (ASB) formation [28,29,30,31,32,33]. ASB forms in THA penetrators (Figure 1), but only occasionally because of specific microstructures consisting of hard 30–40 μm size tungsten grains embedded in a relatively soft matrix. Such a specific microstructure (sometimes called composite) forces the ASB to spread in a zigzag path. However, we suspect that the spreading of ASB would be much easier in THA with finer tungsten grains. The size of tungsten grains is controlled with an LPS parameter (time and temperature) [1,7,15,27,33], but also can decrease with some alloying species (e.g., Re addition [27,34,35,36,37,38,39,40,41,42,43,44]). Therefore, we conducted a preliminary investigation concerning the influence of rhenium on the microstructure and mechanical properties of W-Ni-Fe-Co type heavy alloy. The main goal of the study is to verify if and how much Re addition influences the tungsten grain size?

## 2. Experimental Procedure

**Figure 2:**
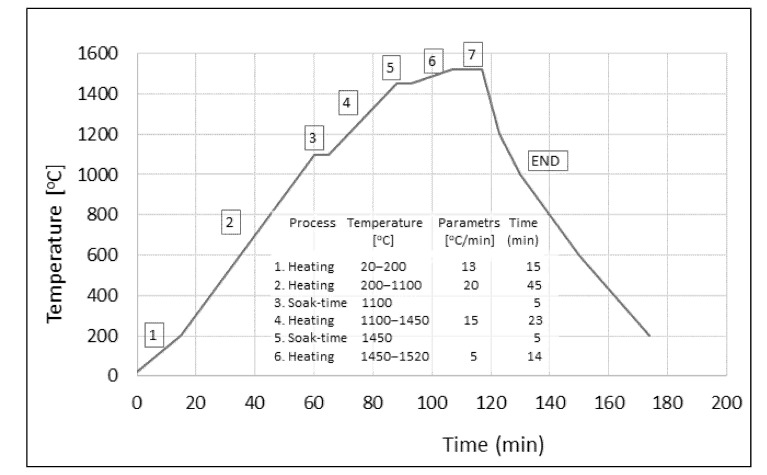
Scheme of single heat treatment: (stage 7) 20 min liquid phase sintering at temperature 1520 °C.

The tungsten alloy W90.8Ni6.2Fe1.2Co1.8 designated as PR-100 was selected for the study. This alloy was “base” alloy; its composition was modified with a small amount of rhenium. The composition of three THA with rhenium selected for the study was calculated this way, to assure the same density of the alloys. Since density of rhenium (20.02 Mg/m^3^) is comparable with tungsten (19.30 Mg/m^3^), the rhenium addition replaced tungsten in the powder mixture. The chemical composition of alloys is provided in Table 1. High purity tungsten, nickel and iron powders were mixed with pure rhenium. The rhenium powder had to be powdered to a size of about 3–10, mechanically in agate mortar. The powder components were mixed, compressed, and then sintered in a hydrogen atmosphere, according to the schedule provided in Figure 2.

Liquid phase sintering was carried out under a hydrogen atmosphere in a Vacum Industries chamber furnace (Vacuum Industries, Inc., Somerville, MA, USA). Proper sintering consisted of a 5 min soak-time at the temperature of 1520 °C (7 stage on the graph, Figure 2). Starting from a temperature of 1100 °C (point END on the graph), the atmosphere was changed from hydrogen to nitrogen. After LPS, the specimens in the shape of cylindrical rods with a diameter of 18 mm and length of 510 mm, were subjected to heat treatment in a vacuum furnace to remove the hydrogen introduced during the liquid phase of sintering. The heat treatment annealing was carried out in a vacuum at 1100 °C for 180 min. In the final step, the rods were removed and flash cooled in water.

First, the density of the alloys were measured using the Archimedes method. The microstructure investigations were carried out using conventional metallography. The observations of the microstructure were carried out on the Nikon Eclipse MA-200 Microscope (Nikon Corporation; Tokyo, Japan), using objectives with a magnification of 50 to 1000×. The typical microstructure photos were digitalized for quantitative analysis. For the qualitative analysis, both longitudinal and transverse cross sections of the specimen were used. The mechanical testing was used for evaluation of tensile strength—R_m_, yield stress—R_p 0.2_, and elongation—A_5_. For the mechanical testing standard, quintuple samples with a diameter of 5 mm were used. The tests were carried out on the Instron model 1115 testing machine (Instron; Norwood, MA, USA) with a 100 kN head and a traverse speed of 1 mm/min.

Except for the standard mechanical testing, a hardness measurement of tungsten grains and the Ni-based matrix were performed. The experiment was carried out on a Future-Tech FM-810 tester (Future-Tech Corp; Tokyo, Japan) under a load of 25 g (dwell time: 15 s). The research was carried out on previously performed metallographic specimens.

The analysis of the chemical composition, in particular of the rhenium content in the tungsten grains, was carried out on a scanning electron microscope (Carl Zeiss Microscopy GmbH, Jena, Germany) by Zeiss Ultra Plus with an attached EDS system (Quantax 400) by Bruker.

## 3. Results

### 3.1. Results of Density Measurements

The results of density testing are provided in Table 2. It follows from Table 2 that the density of all alloys is almost the same and the calculated values equal the calculated one; this means also that the specimens are free of discontinuities and pores.

### 3.2. Results of Mechanical Testing

**Figure 3:**
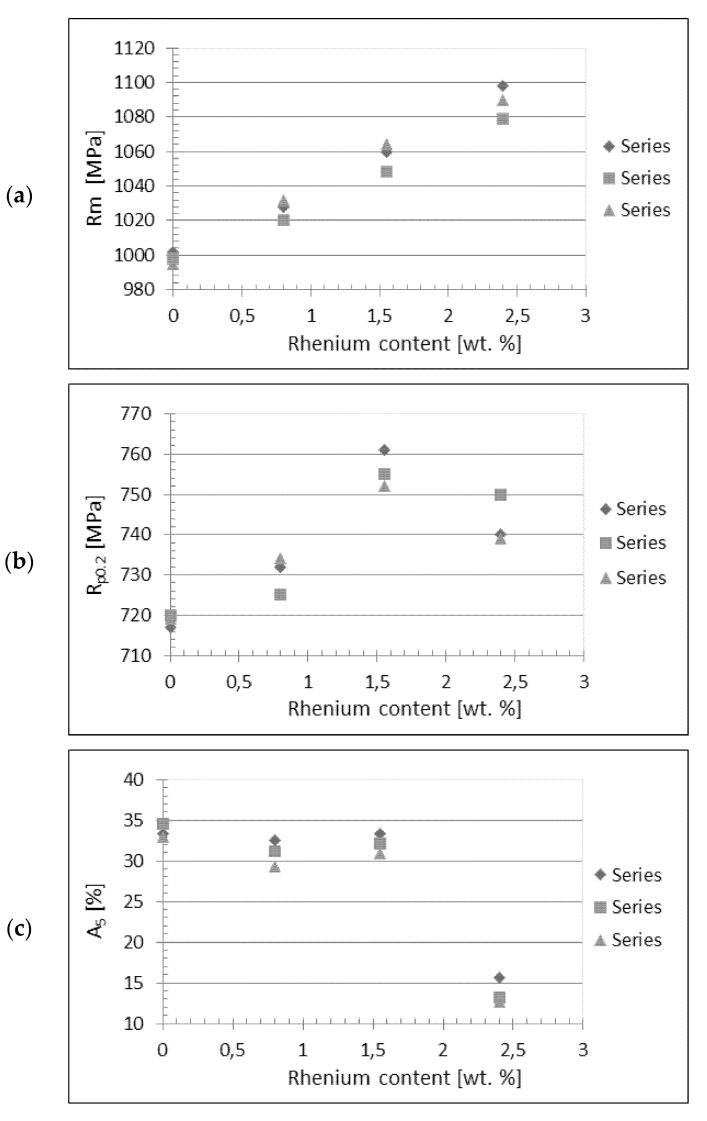
The results of tensile tests as a function of sintering cycles: (**a**) tensile strength, (**b**) yield strength, and (**c**) elongation.

**Figure 4:**
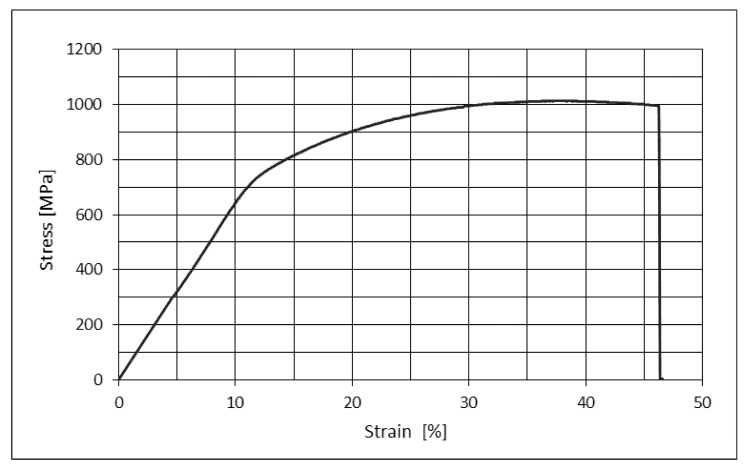
The stress-strain curve for the specimen Re-1 (0.8% Re).

The results of the mechanical testing are presented in Table 3. It is visible from the table that the rhenium addition influences tensile strength, yield stress, and elongation, but this influence is different and depends on the amount of rhenium. Figure 3 displays the graphic illustration of the dependence of tensile strength, ultimate tensile stress, and elongation in the function of rhenium content. Figure 4 displays the stress-strain diagram for the sample with 0.8 wt.% Re.

It is visible from the graphs that the rhenium content increase causes an almost simultaneous increase of tensile strength (Figure 3a). In the case of ultimate stress, the maximum value R_p 0.2_ is observed for 1.5% of Re and then it decreases with the Re increase (Figure 3b). The elongation value is almost constant up to 1.55% Re and then drops drastically for 2.4% Re (Figure 3c). The behavior of elongation with rhenium content was expected on the basis of literature information, where it can be found that an excess of rhenium causes brittleness of tungsten alloys.

### 3.3. Results of Microhardness Measurement

**Figure 5:**
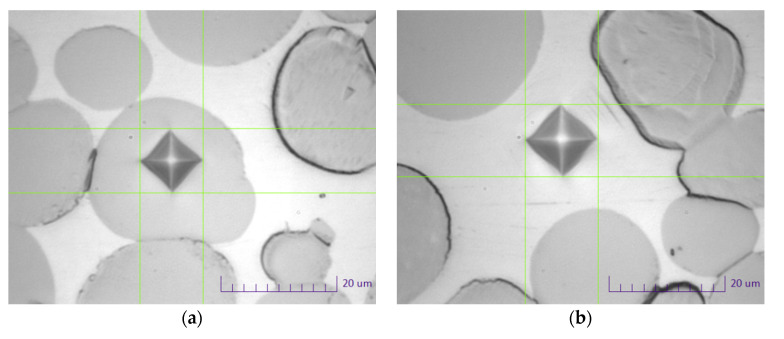
Sample photos of measuring microhardness of tungsten grains (**a**) and matrix (**b**).

The average values of the microhardness measurements of tungsten grains and matrix are presented in Table 4. Figure 5 shows examples of microhardness measurement in the area of tungsten grain and matrix. Average values were obtained by performing 20 measurements in tungsten grains and the matrix.

Larger microhardness scatter (standard deviation) was obtained for the measurement in the matrix. The likely cause of this is with the WHA microstructure; there may be tungsten grains under the thin warp layer. Thus, when performing the matrix measurement, the measurement in tungsten grains is partially carried out. In each of the WHA materials, the microhardness of tungsten grains is higher than the microhardness of the matrix. The microhardness of the matrix and tungsten grains in the alloy without the addition of rhenium (PR-100) is, respectively, 345 and 440 HV 0.025. With an addition of 0.80 wt.% Re (Re-1), no increase in matrix microhardness and a decrease in tungsten grain microhardness is observed. For the alloy with an intermediate amount of rhenium (Re-2), the matrix microhardness increased by approximately 30 HV 0.025 units. The microhardness of the tungsten grains, taking into account the error value, did not change. For the highest rhenium content in the alloy (Re-3), an increase in the matrix microhardness by approx. 45 HV 0.025 units is visible. The microhardness of tungsten grains increases by 30 HV 0.025 units.

### 3.4. Microstructure Observations

**Figure 6:**
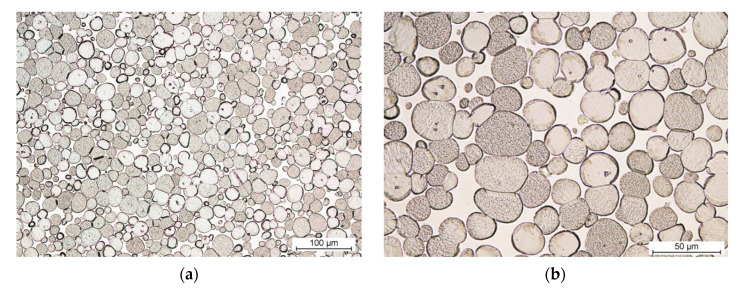

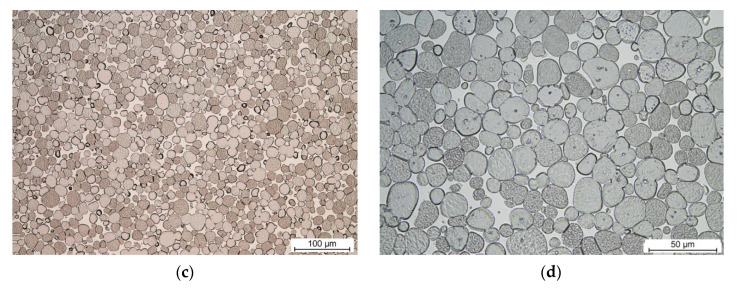
The microstructure of THA: (**a**,**b**) without addition of Re (PR-100), (**c**,**d**) with 2.4 wt.% Re (Re-3), (**a**,**c**) mag. 200×, and (**b**,**d**) mag. 500×.

In Figure 6, the examples of THA microstructure are depicted. The micrographs are specially given at the same magnification to allow immediate comparison of the tungsten grains’ diameter.

The microstructure of all WHA materials is homogeneous, consisting of shaped, elliptical tungsten grains in the matrix of the binding phase. No pore clusters were found in the microstructure.

For each sample, at least 100 measurements of tungsten grains were carried out. Then two of them, the maximum and minimum values, were removed, and the rest were used for statistical evaluation. The results of the tungsten grain size measurement are provided in Table 5. The values provided in Table 5 are the mean diameter converted to the area of the circle.

It follows from Table 5 that the increase of Re content causes the systematic decrease of tungsten grain size.

The average diameter of the tungsten grains in the WHA material without the addition of Re is 33 µm. Grain size reduction in the material with the lowest Re content (0.8 wt.%) is at the level of just a few percent. Tungsten grain size reduction in an alloy with an intermediate Re content (1.55 wt.%.) is 20%. In the WHA material with the highest Re addition (2.4 wt.%), the tungsten grain size was reduced by 40%.

### 3.5. Chemical Microanalysis Tungsten Grains and Matrix

**Figure 7:**
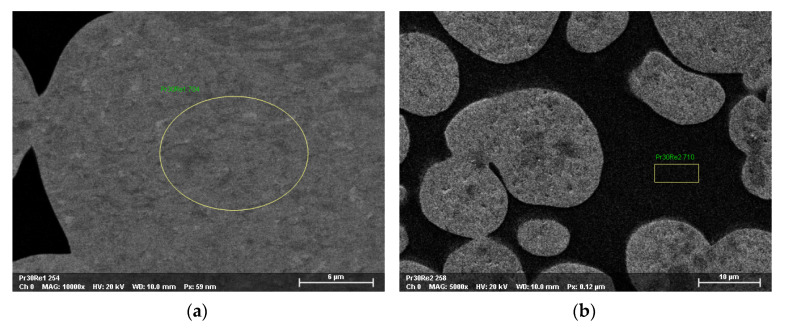
SEM micrograph with marked places of EDS analysis in the area of tungsten grains (**a**) and matrix (**b**).

The microanalysis of studied alloys was performed in a scanning electron microscope equipped with am EDX attachment. The aim of this study was to evaluate the concentration of rhenium in tungsten grains and the matrix. The results of the chemical composition study are depicted in Table 6. Examples of EDS analysis sites are shown in Figure 7.

It is visible from the tables that rhenium occurred either in tungsten grains or the matrix, but the solubility of rhenium in tungsten grains reaching 2.46 wt.% is much higher in tungsten grains than in the Ni-based matrix, where it arrives at 0.82 wt.% only.

The solubility of rhenium in tungsten grains is comparable to the percentage of rhenium in the chemical composition of individual WHA materials.

## 4. Discussion of Results

For many years, work has been carried out to increase the armor penetration by kinetic anti-tank missiles. The armor penetration depth of this type of ammunition depends on many factors, including (at the same projectile velocity) the density and mechanical properties of the penetrator used. One of the directions of development of this type of ammunition is research in the field of modification of the chemical composition and heat and mechanical treatment processes of the penetrator’s construction material, in the direction of obtaining an appropriate metallographic structure ensuring a self-sharpening effect. One of the possible methods of increasing the penetration capacity of the armor by penetration may be by adding rhenium to the WHA chemical composition. Due to its density higher than tungsten, rhenium does not lower the WHA material density, improves strength properties, and decreases the size of tungsten grains in the alloy microstructure. The advantages of rhenium presented above may facilitate the formation and spreading of adiabatic shear bands (ASB) during the target penetration process and the self-sharpening effect.

The Re-W binary system [45,46] indicates the formation of solid solutions between tungsten and rhenium from the temperature of 1500 °C to the melting point of individual elements. The maximum solubility of rhenium in tungsten at the temperature of 3000 °C is 37%. The solubility at 1500 °C is 28%. The solubility of tungsten in rhenium at the temperature of 2800 °C is 20%, and at the temperature of 1500 °C it is 12%.

Rhenium solubility in nickel at 1620 °C is 17.4%. It should be emphasized that the attached system displays equilibrium with the W-Re phase above the temperature of 1500 °C.

The presented results of the analysis of the chemical composition of tungsten grains by the EDS method confirm the formation of solid solutions of tungsten with rhenium. The solubility of rhenium was confirmed both in the binding phase, which was a solution on a nickel matrix, and in tungsten grains. The rhenium content in tungsten grains is comparable to the percentage of rhenium powder added to individual mixtures (Table 2).

In traditional W-Ni-Fe alloys, along with increasing the amount of tungsten, the theoretical density increases, and the amount of matrix decreases. This translates into a change in the microstructure, an increase in the share of low-energy intercrystalline boundaries between the contacting tungsten grains. Increasing the amount of tungsten in the alloy causes an increase in hardness, but when its’ optimal amount is exceeded, the strength, plasticity, and impact properties decrease (e.g., in W-Ni-Fe alloys with a content of more than 96% by weight of tungsten, the values above the mentioned parameters drop significantly [15]). The formation of solid solutions of rhenium and tungsten enables the production of high-density tungsten heavy alloys without losing mechanical properties. The high density of rhenium and the fact of its steaming in the tungsten grains allows it to maintain the optimal amount of the matrix, without increasing the direct contact points between the tungsten grains (called contiguity) and without reducing the strength and impact toughness value.

## 5. Conclusions

Based on the research, it can be concluded that at given LPS parameters:

- Rhenium is dissolved in the tungsten grains to a content of at least 2.4% by mass.
- Rhenium promotes the grinding of tungsten grains. With 1.5 wt.%, changes in the microstructure are visible; there is a marked reduction in the size of the tungsten grains. With the increase in the rhenium content in the alloy, the size distribution of the measured grains decreases.
- Addition of Re has a relatively small influence on tungsten grain hardness (~7%), but causes a visible increase of matrix hardness (~15%).
- The increase of rhenium content increases the porosity. It can be suggested that increased porosity decreases plasticity by increasing the matrix sensitivity on the stress concentration factor. This in turn could explain the drop in elongation in cases with higher (2.4 wt.%) Re content.
- The addition of rhenium increases the strength properties of WHA. The value of tensile strength and proof strength increases with increasing shares of rhenium in WHA.
- The addition of rhenium reduces the plasticity of the WHA. As the rhenium content in WHA increases, the elongation decreases. Initially, for the lower amount of rhenium in WHA (0.8 and 1.55% by weight), the reduction in plasticity is slight—4% for the alloy without the addition of Re. With a higher rhenium content in WHA (2.4%), the decrease in plasticity is significant—nearly 60%.

In future research, we would like to study the influence of Re content on the contiguity parameter. Moreover, we are going to carry out a TEM observation using the thin foil method. In this study, we will concentrate on the very subtle structure of the matrix and the region on the tungsten grain-matrix boundaries. Since we are involved into materials dedicated to military application, we will try to see if and how the rhenium addition influences W-Ni-Co type WHA.

## Figures and Tables

**Table 1:** Chemical composition of the alloys.

Alloy Designation	Chemical Composition (wt.%)				
	W	Ni	Fe	Co	Re
**PR-100**	90.80	6.2	1.2	1.8	-
**Re-1**	90.00				0.80
**Re-2**	89.25				1.55
**Re-3**	88.40				2.40

**Table 2:** Density of alloys.

Alloy Designation	Density (Mg/m^3^)	
	Calculated	Measured
PR-100	17.37	17.35
Re-1	17.38	17.37
Re-2	17.39	17.35
Re-3	17.40	17.35

**Table 3:** The results of mechanical testing.

Alloy Type	Mechanical Properties		
	R_p0.2_ (MPa)	Rm (MPa)	A_5_ (%)
**Pr-100**	717	1002	33.4
	720	998	34.5
	719	995	32.9
**Average values**	**719 ± 2**	**998 ± 4**	**33.6 ± 0.8**
**Re-1**	732	1028	32.5
	725	1020	31.2
	734	1032	29.3
**Average values**	**730 ± 5**	**1027 ± 6**	**31.0 ± 1.6**
**Re-2**	761	1060	33.4
	755	1048	32.1
	752	1064	30.9
**Average values**	**756 ± 5**	**1057 ± 8**	**32.1 ± 1.3**
**Re-3**	740	1098	15.6
	750	1079	13.2
	739	1090	12.6
**Average values**	**743 ± 6**	**1089 ± 10**	**13.8 ± 1.6**

**Table 4:** The results of microhardness measurements.

Alloy Designation	HV 0.025	
	Matrix	Tungsten Grain
**PR-100**	345 ± 15	440 ± 12
**Re-1**	340 ± 15	398 ± 18
**Re-2**	380 ± 21	455 ± 13
**Re-3**	398 ± 23	470 ± 12

**Table 5:** The average tungsten grain size.

Alloy Designation	Re Content (wt.%)	Tungsten Grain Size (μm)
**PR-100**		33.1 ± 4.6
**Re-1**	0.80	32.8 ± 4.6
**Re-2**	1.55	26.1 ± 3.1
**Re-3**	2.40	20.6 ± 3.4

**Table 6:** Results of microanalysis of tungsten grains and matrix.

Alloy Designation	Re Content Mass Norm. (%)		Chemical Composition (wt.%)
	Tungsten Grains	Matrix	
**Re-1**	0.82	0.73	0.80
**Re-2**	1.85	0.49	1.55
**Re-3**	2.46	0.82	2.40

## Data Availability

Not applicable.
